# Properties of Thermoplastic-Bonded Plywood: Effects of the Wood Species and Types of the Thermoplastic Films

**DOI:** 10.3390/polym12112582

**Published:** 2020-11-03

**Authors:** Pavlo Bekhta, Marcus Müller, Ilona Hunko

**Affiliations:** 1 Department of Wood-Based Composites, Cellulose, and Paper, Ukrainian National Forestry University, 79057 Lviv, Ukraine; ilona.hunko@ukr.net; 2 University of Applied Forest Sciences Rottenburg, 72108 Rottenburg am Neckar, Germany; mueller@hs-rottenburg.de

**Keywords:** thermoplastic-bonded plywood, thermoplastic films, bending strength, modulus of elasticity, bonding strength, thickness swelling, water absorption, wood species

## Abstract

There are a lack of proper adhesives that meet the wood industry requirements of being environmentally friendly, low cost, and easy to use; thus, the application of thermoplastic polymers, especially films, is promising. This work expands our knowledge about the possibility of using thermoplastic films for the production of environmentally friendly plywood. The effects of the adhesives type and wood species on the properties of plastic film bonded plywood were studied. Sliced veneers of two hardwoods (birch and beech) and one softwood (spruce) were used in the experiments. Three types of thermoplastic films—low-density polyethylene (LDPE), co-polyamide (CoPA), and co-polyester (CoPE)—were used as an adhesive for bonding plywood samples. Melamine–urea–formaldehyde (MUF) resin was used as a reference. The influence of the type of adhesive and wood species as well as their interaction on the properties of plywood was significant. The lowest bonding strength demonstrated plywood samples bonded by LDPE, and the highest bonding strength in the samples was shown in those bonded by CoPA. A significant difference was found between softwoods and hardwoods in terms of their influence on the physical and mechanical properties of plywood samples. From the obtained data, it follows that softwoods provide much lower values of bending strength (MOR), modulus of elasticity (MOE), and bonding strength than hardwoods. The obtained bonding strength values of plastic-bonded plywood panels ranged from 1.18 to 2.51 MPa and met the European standard EN 314-2 for Class 1 (dry conditions) plywood.

## 1. Introduction

Plywood is one of the most important value-added panel products in the wood industry. In 2018, industrial plywood production in the world reached a record output of 163 mln·m^3^. This was the fastest growth among all wood-based panels (particleboard, oriented strand board OSB, and fiberboard), recording 2%/179% and 1%/105% respectively from 2017/2000 to 2018. The global consumption of plywood grew by 2.5% in comparison to 2017 to reach a record consumption of 161 mln·m^3^ [1]. Currently, different kinds of plywood are manufactured using various types of adhesives, mainly formaldehyde-based adhesives such as phenol–formaldehyde (PF) and urea–formaldehyde (UF). These resins have the advantages of low-cost, high reactivity, excellent strength properties, and ease of use for a wide range of conditions for curing [2]. However, it is well known fact that formaldehyde-based adhesives are not environmentally friendly. One of the main disadvantages of these adhesives is that the production and products from them can release free formaldehyde. Since a significant proportion of plywood products are for indoor applications, the release of harmful volatile compounds, including formaldehyde, is very detrimental to human health [3]. It is well known that formaldehyde is a human carcinogen [4], and this fact compels companies to reduce formaldehyde emission to lower levels.

Many studies have been done to reduce the release of formaldehyde or to replace formaldehyde-based adhesives with more environmentally friendly adhesives [5,6,7,8,9,10,11,12]. However, very often, such adhesives are not widely used in the manufacture of plywood. They are either too expensive, do not provide proper bonding quality, have insufficient durability against different biological agents, or are not water-resistant. 

In addition, at present, plywood factories mainly apply glue on veneers using glue drums. The glue is applied in a liquid state. This process requires a high fluidity of adhesives, and it can cause uneven spreading, uneven thickness of the adhesive line, and as a consequence a deterioration of the bonding strength. Moreover, the operations of mixing and applying the glue are untidy and unpleasant. The ideal plywood adhesive should be of uniform quality and form an adhesive line of uniform thickness [13].

From the other side, it is well known that environmental pollution problems and a shortage of natural resources are becoming important issues. A huge amount of waste plastics (≈6.3 billion tons) are generated every year all around the world, while only ≈9% of them are recycled and most (≈79%) of them are accumulated in the natural environment [14]. Plastic is widely used in many applications, especially in the form of disposable products, such as plastic bags, agricultural plastic film, and greenhouse film. These waste products mainly consist of polyethylene (PE), polypropylene (PP), polystyrene (PS) and polyvinyl chloride (PVC) [15]. Plastic films are materials that are not able to biodegrade rapidly in the environment. Today, recycling waste products by utilizing them in manufacturing processes is very attractive, because it can prevent environmental pollution and lower production costs.

Due to the lack of suitable adhesives to meet wood industry requirements of being environmentally friendly, low cost, and easy to use, the application of thermoplastics (PE, PP, PS, PVC), and their copolymers, is promising [16,17]. The great advantage of such polymers is that the formaldehyde emission of the plywood made by recycled plastics is very low compared to that of ordinary plywood made with urea–formaldehyde resin: the amount of emission is almost zero [18]. Formaldehyde-free wood–plastic plywood has been successfully produced using thermoplastic polymers as wood adhesive [18,19,20,21,22,23,24]. The various thermoplastic polymers were used for veneer bonding: low-density polyethylene (LDPE) [25], high-density polyethylene (HDPE) [17,19,20,24,26,27], PS [21,28], PP [16,23], or PVC [29,30]. The thermoplastic polymers were used for veneer bonding in various forms, such as textile fiber waste (polyurethane, polyamide-6) [16], recycled plastic shopping bags [18,26], or film [17,19,20,22,23,24,27,29,30].

Oh [25] found that low-density polyethylene can be used as an additive in phenol–formaldehyde resin adhesive for bonding radiate pine plywood. Modified HDPE powder through in situ chlorinating graft copolymerization has been successfully used to manufacture exterior plywood [31,32]. LDPE film has also been chosen to develop non-formaldehyde plywood, and the resulting plywood meets the requirement for interior plywood application [33]. Wood plastic plywood composed of veneer and styrofoam was manufactured, and its vibrational properties were investigated by Hu et al. [34].

Hot melts based on HDPE, polyurethane textile fiber waste, and unmodified and modified sulfate lignin adhesives for wood veneer bonding were used by Grinbergs et al. [35]. Cui et al. [18] replaced traditional adhesives with compounds made with recycled plastic shopping bags, mainly composed of PE, PP, PVC, and PS, in order to make hot-melt plywood using various amounts of plastic film, different hot-pressing temperatures, and different hot-pressing times. The results show that the bonding strength of plywood does not increase with increasing amounts of plastic film. The optimum hot-pressing parameters are as follows: 100 g·m^−2^ of recycled plastic, a hot-pressing temperature of 150 °C, and a hot-pressing time of 6 min. 

The results showed that polystyrene wastes can be used in plywood manufacturing as an alternative bonding material for interior uses [21,28]. It was shown that low-pressure pre-heating is a necessary step allowing for increasing the final shear strength of the wood thermoplastic joints and avoiding the indentation of thermoplastic particles to wood [28].

According to the findings of another work [16], the use of HDPE, PP, polyurethane, and polyamide-6 textile fibre waste hot melts for wood veneer gluing guarantees the shear strength of the material that considerably exceeds the adhesive strength of industrial plywood glued with phenol–formaldehyde resins. Moreover, their use would eliminate glue toxicity and considerably improve environmental protection.

Fang et al. [19] demonstrated that it is possible to manufacture environmentally friendly plywood directly bonded with unmodified plastic film. The main component of the plastic film used in the experiments was HDPE with a thickness of 0.05 mm per layer. The results showed that the highest strength was obtained under hot pressing conditions (pressure, 0.7 MPa; temperature, 160 °C; time, 1 min·mm^−1^; and film dosage of two layers), which was comparable to that of commercial UF-bonded plywood. The pressing temperature had a notable effect on adhesive penetration and bonded joints formed. In another work [27], it was found that the HDPE film dosage positively affects the properties when ranging from 61.6 to 246 g/m^2^. The performance of these plywood panels was comparable to those of plywood made with commercial UF resins.

The effect of various surface treatments on the adhesion between wood veneers laminated with a thin PVC film has been investigated using XPS, contact angle, and surface tension measurements, and a lap shear-test [29]. Wood veneers were treated with silanes (amino and chloro), phthalic anhydride, and maleated polypropylene for surface modification. The adhesion between PVC and wood veneer laminates was significantly improved when wood veneers were treated with amino-silane, while no improvement was observed for the other adhesion promoters.

To improve the interfacial adhesion between the wood veneer and HDPE film, silane A-171 was used to treat the surface of poplar veneer by spraying [36]. The shear strength and water resistance of plywood are greatly improved thanks to the silane surface modification. When one layer of HDPE film was used as an adhesive, it caused a 293.2% increase in shear strength, and a 34.6% and 40.8% reduction in water absorption and thickness swelling, respectively. In addition, the wood failure also increased from 5% to 100% due to the silane modification.

In another work [37], to improve the interfacial adhesion between wood veneer and HDPE film, wood veneer was thermally modified in an oven or chemically modified by vinyltrimethoxysilane. The results showed that both modifications reduced veneer hydrophilicity and led to an enhancement in shear strength, wood failure, and water resistance of the resulting plastic-bonded wood composite. However, the strength of silane-treated plastic-bonded wood composite was still much lower than thermosetting resin-bonded composites at higher temperatures. Moreover, this chemical-based method was limited in its industrial use because of high modification costs and relatively complicated processing.

The use of thermoplastic film as an adhesive for the bonding of veneer is the most promising. Apart from the fact that the plastic film is formaldehyde-free, its use also has several other advantages compared with using liquid adhesives. Dry adhesive film is simpler to apply than wet adhesives; all of the untidy and unpleasant mixing and spreading operations in wet gluing are wholly removed from the plywood factory by the use of dry adhesive film. The dry adhesive film contains in each square meter of surface precisely the same quantity of adhesive, equal quality, uniform composition, exactly the same bond strength, and the same standard thickness [13].

Plywood is produced from softwood and hardwood species, and the species used in its manufacture determine the physical and mechanical properties of the plywood [38]. However, no information was found in the literature regarding the impact of various wood species and different type of thermoplastic polymers, particularly co-polyamide and сo-polyester on the properties of plastic-bonded plywood. Therefore, the purpose of this study was to obtain a better understanding of the bonding process of plastic plywood when using various wood species and different types of thermoplastic polymers.

## 2. Materials and Methods

### 2.1. Materials

Sliced veneers of two hardwood species of birch (*Betula verrucosa* Ehrh.) and beech (*Fagus sylvatica* L.) and one softwood species of spruce (*Picea abies*) with a thickness of 3.0 mm and a moisture content of 8 ± 2% were used in the experiments to evaluate the effect of wood species on the ability of veneer sheets to bond with thermoplastic film. Veneer sheets with a dimension of 470 mm × 117.5 mm were made in the laboratory conditions. To minimize the influence of wood structure defects on the results of the experiment, the veneer sheets were selected and evaluated for the production of plywood panels. The veneer sheets were visually checked, and sheets without shocks, cracks, curling, and colors of more or less uniform thickness were selected. Observation of the wood appearance did not show any visible defects. 

In this study, liquid adhesive and dry thermoplastic films were used. Three types of thermoplastic films: low-density polyethylene (LDPE), co-polyamide (CoPA) and co-polyester (CoPE) were used for bonding plywood samples. The virgin polymer films LDPE and both CoPA and CoPE were purchased from the “dm-folien GmbH”, Reutingen, Germany and “Gerlinger Industries GmbH”, Netzschkau, Germany, companies respectively. The plastic film was cut into the same dimensions as the veneer samples. The characteristics of the films are shown in Table 1. Melamine–urea (MUF) resin Prefere 4546 (Dynea AS, Lillestrøm, Norway) with a 64–66% solid content, a viscosity of 3500–6000 mPa.s, a density of about 1.3 g/cm^3^, and a pH value of 9.0–10.0 was also used for the comparison. Prefere 5022 (Dynea AS, Lillestrøm, Norway) was used as a hardener. MUF adhesive was prepared in accordance with the recommendation of producer: the mixing was done with a stirrer and the mixing ratio amount was 100:50 (resin:hardener). No water was added. 

### 2.2. Manufacturing of Plywood Samples

The moisture of the veneers can cause the formation of bubbles and poor bonding, especially when thermoplastic films are used. Therefore, the veneer sheets were oven-dried before bonding. The oven-drying of veneer also helped prevent (avoid) deformation of the veneer sheet during hot pressing, which was caused by the release of moisture from the wood under pressure and high temperature. 

Three-layer plywood samples with dimensions of 470 mm × 470 mm were prepared. Instead of traditional MUF adhesive, a plastic film was used as an adhesive for manufacturing the plywood samples. It must be noted that the assembling procedure follows those used in the plywood production, the principle of which is that the veneers were laid with the directions of the fiber perpendicular to each other. Thermoplastic films mainly work as the glueline connecting between two veneers. Two sheets of plastic film were incorporated between the adjacent veneer sheets. 

The veneer assemblies were pressed at the pressing pressure of 0.8 MPa and temperature of 150 °C for 5 min (Table 2). Plywood samples bonded with MUF adhesive were made at the same pressing conditions as the plastic film-bonded samples, and the adhesive spread rate was 200 g/m^2^ based on wet mass. The adhesive mixture was applied to the surface of the veneer by hand using a hand roller spreader, and the open assembly time was about 5 min. Two sheets of plastic film were incorporated between the adjacent veneer layers. The dosage of plastic LDPE, CoPA, and CoPE films at thicknesses of 0.06, 0.04, and 0.05 mm for two sheets of film between adjacent veneers equals 114, 88, and 125 g/m^2^, respectively. For LDPE, CoPA, and CoPE films this was 43%, 56%, and 37.5% less, respectively, than for MUF adhesive.

The pressing temperature depends on the adhesive. As can be seen from the Table 1, the melting points of the investigated films are at a lower threshold of the hot pressing temperature. The hot pressing temperature should exceed the melting point to provide a good fluidity of the molten film and its good penetration into the cavities (voids) of wood. On the other hand, the temperature should not be too high, which may result in a degradation of the wood veneer and also the decomposition of the thermoplastic polymer. Therefore, the plywood samples were pressed at a temperature of 150 °C. After hot pressing, the plywood samples were subjected to a cold-press stage that was performed at room temperature, which was performed to release internal stresses and reduce the warping of plywood. Three replicate panels were manufactured for all the conditions and control.

### 2.3. Panel Testing

**Figure 1:**
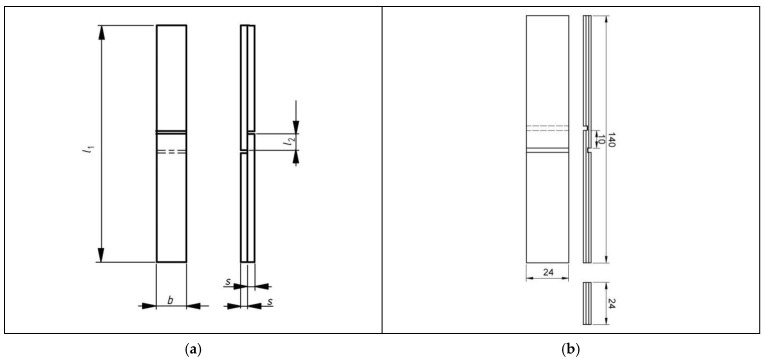
Bonding strength test samples: (**a**) for the bond strength in longitudinal tensile shear strength according to EN 302 (*l*_1_—length of the test specimen; *l*_2_—length of the overlap (length of the surface to be tested); *b*—width of the test specimen (width of the surface to be tested); *s*—thickness of the veneer) (**b**) for the shear strength according to EN 314-1.

The physical and mechanical properties of plastic film-bonded plywood were compared with melamine–urea–formaldehyde (MUF)-bonded plywood and relevant plywood standards. The thickness, density, bending strength (MOR), modulus of elasticity (MOE) in bending, bonding strength, water absorption, and thickness swelling of plywood samples were determined according to the standards [39,40,41,42,43,44,45]. During the experiment, all plywood samples were conditioned prior to testing for one week at 20 ± 2 °C and 65 ± 5% relative humidity. After that, the panels were cut to extract test samples according to the standard requirements. For the bonding strength test, one-half of the samples were tested according to EN 302-1 [42] by determining the bond strength in longitudinal tensile shear strength (Figure 1a) and the other half according to EN 314-1 [43] and EN 314-2 [44] standards by determining the shear strength (Figure 1b). Wood failure percentage was also determined according to EN 314-1 [43]. For the tensile shear strength test, the test pieces were immersed in cold water for 4 days at (20 ± 5 °C) and after that were tested in wet condition (treatment A2). The shear strengths were measured after pre-treatment for bonding class 1—dry conditions—plywood test pieces were immersed in water at (20 ± 3 °C) for 24 h. Ten samples were used for each variant shear/tensile strength mechanical testing. 

MOR and MOE tests were carried out for plywood panels manufactured according to EN 310 [41] standard. Twelve samples were used for the evaluation of plywood MOR and MOE. 

Dimensional stability in the form of thickness swelling (TS) and water absorption (WA) of the samples were determined according to a water-soaking test based on EN 317 [45], using test pieces of dimension 50 mm × 50 mm. They were immersed in distilled water for 2 and 24 h. After this time, the test pieces were removed from the water, weighed, and the thickness was measured. The samples were weighed to the nearest 0.001 g and measured to the nearest 0.01 mm immediately. Six replicate samples were tested for each type of plywood panel. The percent change from the original thickness represents the TS, and the percent weight change from the original weight represents the WA.

### 2.4. Statistical Analysis

Statistical analysis was conducted using SPSS software program version 22 (IBM Corp., Armonk, NY, USA). Analysis of variance (ANOVA) was performed on the data to determine significant differences at the 95% level of confidence. Duncan’s multiple range test was used to determine the significant difference between and among the groups.

## 3. Results and Discussion

### 3.1. Thickness and Density of Plywood Samples

To manufacture of plywood panels, it is important to choose such pressing parameters to prevent a significant thickness loss of panels. ANOVA analysis showed that the type of adhesive and wood species used significantly affects the thickness and density of the plywood samples (Table A1 in Appendix A). 

The average values of the thickness and density of plywood samples are given in Table A3. The European standard EN 315 [39] specifies tolerances of unsanded plywood panels for a nominal thickness of 9 mm as −0.67 mm (min) and +1.07 mm (max); i.e., the thickness of the finished unsanded plywood panels should be in the range 8.33–10.07 mm. In this study, the values of plywood thicknesses were 9.06 ± 0.04 mm for panels made using MUF adhesive, and 8.97 ± 0.18, 8.91 ± 0.15, and 8.89 ± 0.21 mm for panels made using LDPE, CoPA, and CoPE films (Table A3), respectively, and they did not go beyond tolerances for unsanded panels in accordance with this standard. It can be seen that the average thickness of plastic film-bonded plywood panels made was smaller than the thickness of control plywood using MUF adhesive (Table A3).

The smallest thickness (8.89 mm) and density (609.0 kg/m^3^) had CoPE-bonded plywood samples. The largest thickness (9.06 mm) and the highest density (627.5 kg/m^3^) had plywood samples made using MUF adhesive. The difference in the thickness values of CoPA- and CoPE-bonded plywood samples was insignificant based on Duncan’s test. The average density of plastic film bonded plywood panels made was slightly lower than the density of control plywood using MUF adhesive (Table A3). This can be explained by the fact that less moisture was brought with the plastic film, in comparison with MUF adhesive, into the veneer package, and such a package, in turn, is less densified. Moreover, MUF adhesive has a highest density among the investigated adhesives. Other authors [17] also obtained similar results. Between the LDPE and CoPA, MUF, and CoPA as well as LDPE and CoPE adhesives, there is no significant difference based on Duncan’s test in the effect on the density of the plywood samples (Table A3). Conversely, Bal and Bektas [38] studied plywood produced from eucalyptus, beech, and poplar veneers using MUF, UF, and PF adhesives and found that the effect of adhesive type on density was significant. They explained this by the heterogeneity of veneer density among the groups and the amount of extender. Shukla and Kamdem [46] noted that the contribution of the density of the adhesive in the increase of the density of laminated materials is not negligible and should be taken into account in the manufacturing parameters of these materials.

It was also found that differences in the thickness and density values of the panels made using beech, birch, or spruce veneers were significant (*p* ≤ 0.05) based on Duncan’s test (Table A3). The density of veneers significantly affected the thickness and density of plywood samples. Naturally, the smallest and largest thickness and density were observed in the spruce and beech plywood samples, respectively. This pattern is due to the higher density of beech compared to spruce veneers. Nevertheless, according to the *F* values (Table A1), it can be seen that in the ranking from highest to lowest, the wood species has the greatest influence on the thickness and density of the plywood samples, which is followed by the type of adhesive and its interaction with the thickness, the interaction between the type of adhesive and wood species, and the effect of the type of adhesive on the density of the samples.

### 3.2. Bending Strength and Modulus of Elasticity in Bending of Plywood Samples

**Figure 2:**
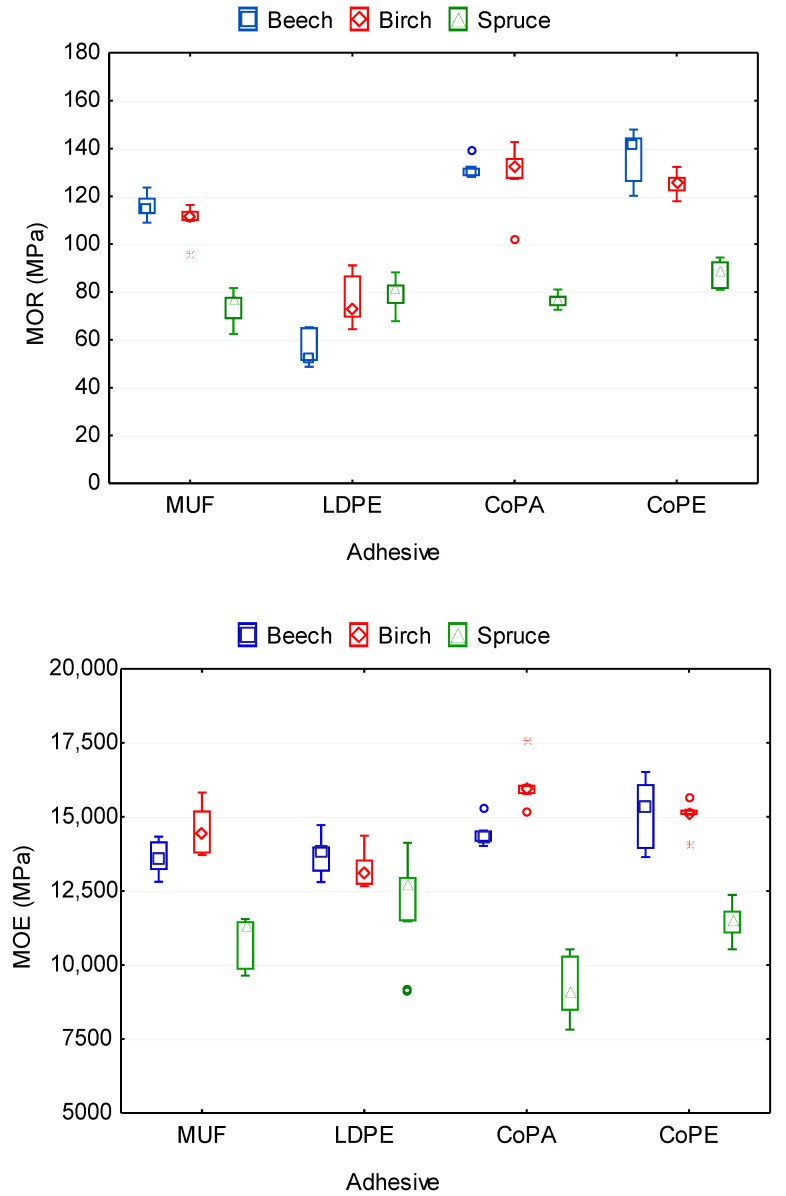
Bending strength and modulus of elasticity in bending of plywood panels bonded with different types of adhesive and using various wood species.

**Figure 3:**
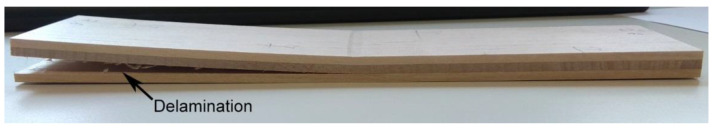
Delamination in the low-density polyethylene (LDPE)-bonded plywood samples during the determination of bending strength.

Figure 2 shows an effect of the type of adhesive and wood species on MOR and MOE of plywood samples. As shown by ANOVA analysis (Table A2), the type of adhesive and wood species significantly affect the MOR and MOE. The highest values of MOR 112.2 and 116.1 MPa had samples bonded by CoPA and CoPE, respectively. Moreover, both these films had the same insignificant effect (*p* > 0.05) on MOR according to Duncan’s test; they belong to the same homogeneous subset (Table A3). The smallest MOR value of 72.3 MPa was found in plywood samples bonded by LDPE. One of the reasons could be the lowest adhesive spread of this film in comparison with other investigated films CoPA and CoPE and MUF adhesive. According to previous studies, the low-adhesive spread may reduce the physical–mechanical properties of plastic-bonded plywood [47]. During bending, plywood veneer and plastic film layers deform similarly. The bending stresses concentrate on the weaker border surface, and LDPE-bonded plywood fracture takes place through the delamination of layers (Figure 3). This can diminish the bending strength of plywood.

The effect of the type of adhesive on MOR and MOE can be explained by the different properties of MUF and thermoplastic films as well as the differences among films themselves. However, it is quite difficult to compare them with each other in terms of their effect on the properties of plywood samples, including MOR and MOE, because they had the different thicknesses and densities (Table 1), and their spread rate was also different.

In several studies, different results were obtained concerning the effect of adhesives type on the mechanical properties of veneer-based products. In some of the research, it was noted that the type of adhesive affected MOR and MOE [38,48]. Conversely, Shukla and Kamdem [46] conducted a study of the bending properties of laminated veneer lumber (LVL) made of yellow poplar and found that the adhesives had an insignificant effect. Özalp et al. [49] studied the effects of several adhesives on MOR and MOE, including polyvinyl acetate, polyurethane, and epoxy, and they also found no statistical differences. 

The wood species affect MOR differently. The highest values of MOR 109.4 and 109.9 MPa were found in plywood samples made using hardwood species—birch and beech, respectively. The smallest MOR 79.5 MPa had samples made using spruce veneer. This can be explained by the fact that hardwoods, having a higher density and better mechanical properties than softwoods, provide higher values of MOR. Birch and beech are similar in their physical and mechanical properties, and therefore, the difference between values of MOR for samples made using these wood species was insignificant (*p >* 0.05) (Table A3). The MOR of beech, birch, and spruce solid woods are 104, 110, and 85 MPa, respectively [50]. Thereby, in this study, the plywood samples showed practically the same bending strength as the solid wood. Similarly, Aydın et al. [48] also found that the effect of veneer wood species on some physical and mechanical properties of LVL was statistically significant.

According to the *F* values (Table A2), it can be seen that, in the ranking from highest to lowest, the type of adhesive (*F* = 137.170) has the greatest influence on MOR of the plywood samples; after that, wood species (*F* = 129.792), and finally, an interaction of type of adhesive and wood species (*F* = 41.685). As follows, an interaction between the type of adhesive and wood species also affects significantly on MOR. As can be seen from Figure 2, the plywood samples bonded by CoPA, CoPE, and MUF had the highest values of MOR for beech, birch, and spruce, in the ranking from highest to lowest. The opposite was observed for samples bonded by LDPE. For these samples, the highest values of MOR were found for spruce, which was followed by birch and beech. Moreover, the difference between values of MOR for LDPE-bonded samples using spruce and birch was insignificant. For LDPE-bonded samples, the generated delamination crack longitudinally extended through the sample (Figure 3), and this significantly reduced the maximum failure load.

The opposite pattern was observed for MOE; the greatest effect on MOE showed wood species (*F* = 112.275), after that, an interaction of type of adhesive and wood species (*F* = 9.382), and finally, the type of adhesive (*F* = 3.124). The differences between the values of MOE for plywood samples bonded by LDPE, MUF, and CoPA as well as for samples bonded by CoPA and CoPE were insignificant (Table A3). Similarly as for MOR, the largest values of MOE were found for CoPA and CoPE-bonded plywood samples, and the smallest was found for LDPE-bonded samples. Moreover, the lowest MOE was found in spruce plywood samples, and the highest MOE was found in birch samples. All investigated thermoplastic films make plywoods more flexible without simultaneously decreasing the modulus of elasticity (Figure 2). Similarly, Shukla and Kamdem [46] also found that LVL made with thermoplastic resin is less rigid and more plastic than the LVL made with thermosetting adhesive. An increase of flexibility of plywoods glued with the investigated thermoplastic films simplifies the production of different bended constructions from plywoods [16].

A significant difference was found between softwoods and hardwoods in terms of their influence on MOR and MOE. From the obtained data, it follows that softwoods provide much lower values of MOR and MOE than hardwoods. It is known that hardwoods have a higher density than softwoods [51]. The density of wood is a function of both the thickness of the cell wall and the cell cavity. There is a good correlation between the strength and density of wood; thus, density is the best prediction of wood strength. In fact, mechanical properties within a species tend to be linearly, rather than curvilinearly, related to density [52]. 

### 3.3. Bonding Quality of Plywood Samples

**Figure 4:**
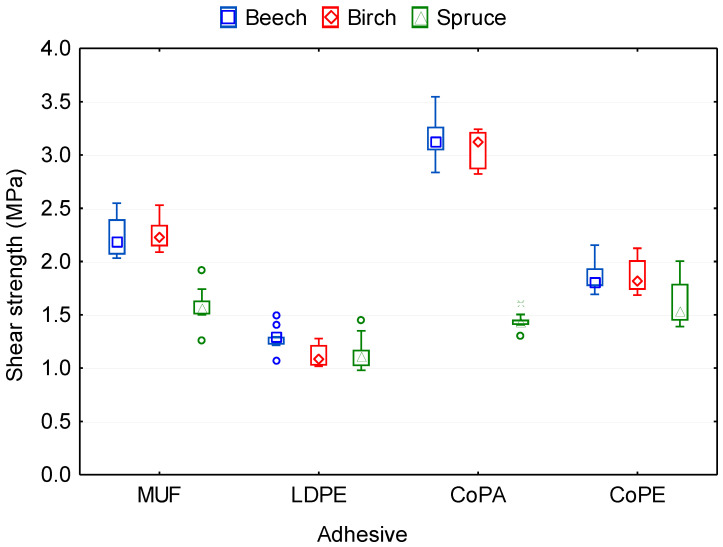

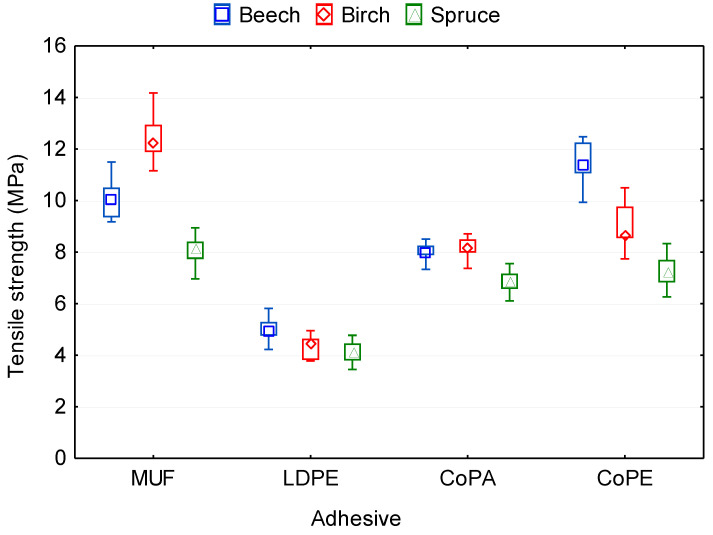
Bonding strength of plywood panels bonded with different types of adhesive and using various wood species.

One of the main properties used to assess the bonding quality is the shear strength of plywood (ShS) according to EN 314 standards [43,44] and the bond strength in longitudinal tensile shear strength (TSS) according to EN 302 standard [42]. ANOVA analysis showed (Table A2) that the type of adhesive and wood species significantly affect both of these properties. All of the shear strength values of thermoplastic plywood panels were higher than 1 MPa (Figure 4, Table A3), which was determined according to EN 314-2 [44]. According to the standard EN 302-1 [42], beech, birch, and spruce samples satisfied the requested minimum 6 MPa of TSS (Figure 4, Table A3) for the A2 condition for the MUF, CoPA, and CoPE adhesives used; LDPE samples were not able to reach the 6 MPa threshold value.

The lowest shear strength of 1.18 MPa demonstrated plywood samples bonded by LDPE, while the highest shear strength of 2.51 MPa was demonstrated in the samples bonded by CoPA. The CoPA film provided 53.1%, 29.1%, and 19.6% higher shear strength values than LDPE, CoPE, and MUF, respectively. The highest TSS 10.17 MPa was observed in control samples bonded by MUF, while it was lower in CoPE (9.29 MPa) and CoPA (7.70 MPa) samples, and the lowest TSS (4.52 MPa), as in the case of a shear strength test according to EN 314, was found in samples bonded by LDPE. These results agree with the analysis of Kajaks et al. [16], who found that the adhesion strength of samples glued with polyamide can be even as high as 10 MPa. However, this tensile shear strength value of plywood samples was obtained at a much higher pressing pressure (2 MPa) and temperature (200–230 °C) than in our study. The percentage of wood failure varies from 0 to 5% for LDPE-bonded samples (Table 3), which is associated with lower ShS and TSS values. In general, strong and durable bonds give high wood failure and fracture deep into the grain of the wood [53]. 

The smallest bonding strength of LDPE-bonded plywood samples could be explained by the lowest adhesive spread rate for this film. During the pressing process, the molten LDPE film was pressed into the vessels and cracks in the veneer, and less molten film remained between the adjacent sheets of the veneer, reducing the bonding strength. The delamination occurred at the boundary between the film and wood (Figure 3). The pressing temperature of 150 °C contributes to the melting of thermoplastic films, which provides better fluidity and allows for thermoplastic to be more evenly distributed. In turn, this creates better conditions for the penetration of molten thermoplastic into the veneer cavities and accordingly creates better conditions for the formation of mechanical locks. Accordingly, this contributes to the increase in the bonding strength [17]. However, on the other hand, it should be taken into account that temperatures higher than 160 °C made the mechanical interlock worse and gave poorer strength to the plywood [17,18,24]. It is obvious that at both high temperature and prolonged pressing time, the PE film is subjected to decomposition and fracturing, so that the rate of increase in the bond strength decreased [17]. Cui et al. [18] concluded that the optimal parameters were a hot-pressing temperature of 150 °C and a hot-pressing time of 6 min.

SEM photographs showed the poor adhesion between veneers and plastic polymer [47]. There are many cavities and voids, which were due to the low interface compatibility between hydrophobic plastic and hydrophilic veneer. Since the films are hydrophobic and the wood is hydrophilic, the consumption of the film, its filling of wood cavities, and the formation of mechanical locks will play an important role in the formation of bonding strength [54]. The adhesion is determined by the mechanical coupling forces arising between the substrate and the polymer as a result of overlapping of the irregularities of wood surface by polymer molecules [54,55]. The amount of mechanical interlocking of the thermoplastic adhesive and the wood surface is dependent on the processing details of the adhesive joint, such as the porosity of the wood surface, viscosity of molten adhesive, applied pressure, and the processing duration. The processes, which kept the thermoplastic polymer molten at the surface of the wood for a longer time, higher temperature, and higher pressure achieved much better interlocking than the short process cycle [55]. Other authors also indicated the lack of chemical bonds between the plastic films and the surface of wood [17,20,24,27,55,56,57]. Goto et al. [56] indicated that the anchoring effect of polypropylene, which had penetrated into various wood elements and spaces in the veneer, contributed dominantly to the gluability. Based on microscopic analysis of the surface and bonding line of plywood, interlocking between veneers by the penetration of a thermoplastic (polypropylene and polyethylene) film into inner and cracks were observed by several authors [54,57,58]. From the results of the microscopic analysis, Kajaks et al. [54] concluded that the polymer adhesive has been pressed into the surface of wood at a depth of 40–50 mm. 

Among the investigated wood species, the highest bonding strength values demonstrated the plywood samples from birch and beech veneers, 2.10 and 2.16 MPa for the shear strength test and 8.58 and 8.40 MPa for the longitudinal tensile shear strength test, respectively. The difference between the values of bonding strength (ShS and TSS) in the samples from birch and beech veneers was insignificant based on Duncan’s test (Table A3). The shear strength and tensile shear strength of the samples from spruce veneer were the smallest 1.40 and 6.62 MPa, respectively. This can be explained by the mechanical properties of the veneers used and anatomical features of the investigated wood species. The effect of wood species on the shear strength is well known, and some other studies have noted that high-density wood species have higher shear strength values. The shear strength values of plywood panels that have greater veneer densities are greater than those for panels that have lower veneer densities [38,59]. Öztürk et al. [60] also found that the panels produced using polyethylene gave shear strength values for hardwoods (beech and alder) better than for softwoods (Scots pine). In addition, it is well known that surface roughness affects the bonding quality of veneer-based products. In our previous study [61], it was found that softwoods (pine) have higher surface roughness than hardwoods (beech and birch). The relationship between surface roughness and some of the properties of plywood is well known [62,63,64]. Rough veneers reduce contact between the layers, resulting in a weak glueline and low strength properties of the plywood [62,63]. Moreover, the surface roughness of the veneer plays an important role in the depth of penetration of adhesive into the veneer, the uniform distribution of adhesive, as well as improvement in the bonding quality between veneer sheets. The layer of adhesive must be of uniform thickness for high bonding quality. 

The bond strength in longitudinal tensile shear strength was much higher than the shear strength of the samples. The TSS and ShS values differ by more than three times. In the TSS test, two sheets of veneer are glued with the same longitudinal direction of the fibers and as a result during the test are stretched along the fibers. It is known that the tensile strength along the fibers of solid beech, birch, and spruce wood is quite high and is 124, 137, and 109 MPa, respectively [50]. In the ShS test, the central sheet of veneer is stretched across the wood fibers. Two adjacent sheets of veneer are glued with a mutually perpendicular direction of the fibers. The tensile strength across the fibers of solid wood for beech, birch, and spruce is 8.5, 6.5, and 3.5 MPa, respectively [50].

### 3.4. Water Absorption and Thickness Swelling of Plywood Samples

**Figure 5:**
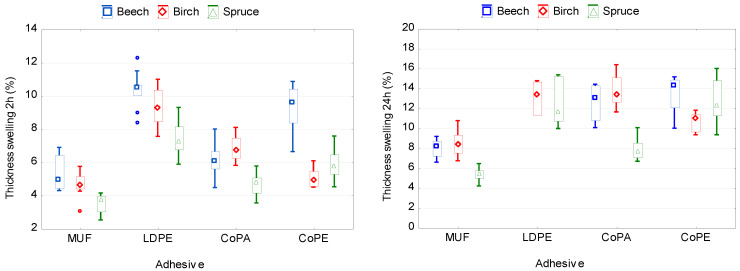
Thickness swelling of plywood panels bonded with different types of adhesive and using various wood species.

**Figure 6:**
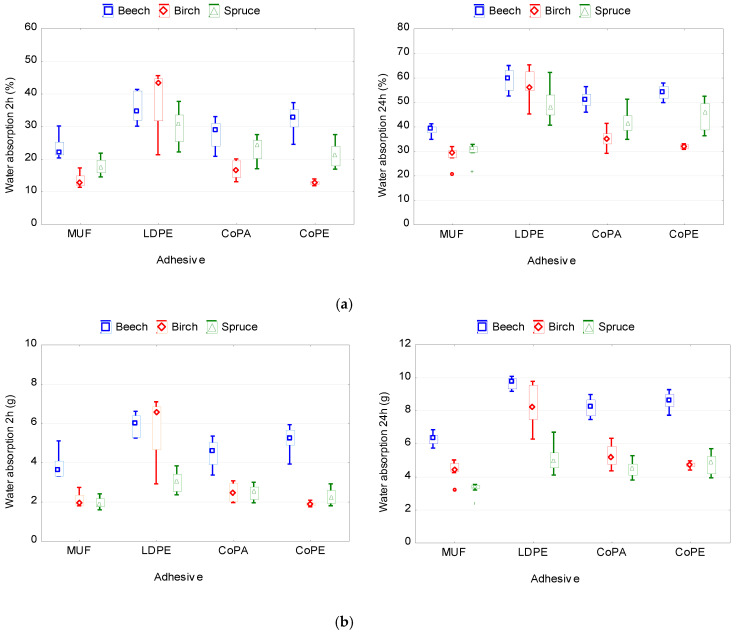
Water absorption of plywood panels bonded with different types of adhesive and using various wood species: (**a**) in percent; (**b**) in absolute values.

Figure 5 and Figure 6 show the influence of the type of adhesive and wood species on the water absorption and thickness swelling of plywood samples. According to the ANOVA analysis, the both variables significantly affect WA and TS (Table A1). 

The lowest values of TS and WA after 2 and 24 h of soaking in water were observed in plywood samples bonded with MUF adhesive. This can be explained primarily by the formation of chemical bonds between the adhesive and the wood. In contrast, the higher values of TS and WA in samples bonded with thermoplastic films are explained by their hydrophilic properties and inability to form chemical bonds with wood. Among the investigated films, the lowest values of TS and WA were shown by the CoPA and CoPE films. The LDPE film showed the worst TS and WA. One of the reasons was the lowest consumption of LDPE film, and as a consequence, an insufficient filling of wood cavities with molten polymer. The consumption of CoPA and CoPE films was higher compared to the consumption of LDPE film.

Moreover, it was found that the type of adhesive has a stronger effect on TS (24 h) and WA (24 h) than wood species (Table A1). This is in good agreement with the known data on the effect of the amount of adhesive on the thickness swelling and water absorption of wood materials such as particleboard or medium-density fiberboard [65]. 

The lowest TS (24 h) was observed in samples made of spruce veneer (9.63%), while the highest TS (24 h) was in samples made of birch or beech veneer (11.46% and 11.20%, respectively). The values of TS (24 h) for birch and beech veneer do not differ significantly (*p >* 0.5). Another pattern was observed for WA (24 h). The lowest WA (24 h) was recorded in samples made of birch veneer (35.89%), there was more in samples of spruce veneer (41.75%), and the highest was in samples made of beech veneer (48.65%).

The obtained results are explained by the differences in the anatomical structure of hardwoods and softwoods. Unlike beech and birch, spruce wood contains more natural resins. These resins reduce the water absorption of the wood. In addition, because spruce has a low density, the amount of bound moisture in the cell walls is also lower than in hardwood species. Bound water molecules, condensing in the cell walls, fall into the spaces between the microfibrils. This causes a thickening of the cell walls and, as a consequence, swelling of the wood. Moreover, the lignin content of hardwoods is usually in the range of 18–25%, whereas the lignin content of softwoods varies between 25 and 35% [66]. Due to the higher lignin content, spruce wood has a more hydrophobic character than the used hardwoods.

## 4. Conclusions

It was found that properties of formaldehyde-free plastic bonded plywood panels were significantly influenced by the type of adhesive and wood species. From the point of view of bonding strength, the CoPA film can be recommended for the bonding of beech and birch, and CoPE film can be recommended for the bonding of spruce. For spruce, the use of CoPE film provides a bonding strength comparable to the bonding strength provided when using an MUF thermosetting adhesive. The lowest shear strength was demonstrated by plywood samples bonded by LDPE. Among the investigated wood species, the highest bonding strength values were demonstrated by the plywood samples from birch and beech veneers. The TSS and ShS values differ by more than three times. The softwoods provide much lower values of MOR, MOE, ShS, and TSS than hardwoods. 

The average thickness and density of the plastic film-bonded plywood samples made was slightly lower than those of MUF-bonded samples. The smallest and largest thickness and density were observed in the spruce and beech plywood samples, respectively. The lowest values of TS and WA were observed in MUF-bonded plywood samples. Among the investigated films, the lowest values of TS and WA were demonstrated by CoPA and CoPE films. The plywood samples made of spruce veneer had lower values of TS than samples made of hardwoods veneers.

The findings of this study provide useful information necessary for optimizing the environmentally friendly plywood manufacturing process when thermoplastic films are used.

## Figures and Tables

**Table 1:** Characteristics of the thermoplastic films.

Type	Thickness (mm)	Density (g/cm^3^)	Melting Temperature (°C)
LDPE	0.06	0.95	105–115
CoPA	0.04	1.10	120–130
CoPE	0.05	1.25	120–130

**Table 2:** Manufacturing conditions of plywood.

Test No.	Manufacturing Conditions				
	Adhesive Type	Wood Species	Pressing Pressure (MPa)	Pressing Temperature (°C)	Pressing Time (min)
1	MUF, LDPE, CoPA, CoPE	Beech, birch, spruce	0.8	150	5

**Table 3:** Wood failure percentage of plastic-bonded plywood samples.

Adhesive	Wood Species	Percentage of Wood Failure (%)	
		Shear Strength Test	Tensile Shear Strength Test
MUF	Beech	92	99
	Birch	77	100
	Spruce	97	99
LDPE	Beech	0	0
	Birch	0	2
	Spruce	0	5
CoPa	Beech	78	64
	Birch	82	79
	Spruce	99	100
CoPE	Beech	4	72
	Birch	14	90
	Spruce	88	98

## Appendix A

### Statistical Analysis

The influence of the type of adhesive and wood species on the physical and mechanical properties of plywood was analyzed using ANOVA analysis. The results are summarized in Table A1 and Table A2. Duncan’s test results for the selected properties of plywood panels are presented in Table A3. The observations made in this study and the results of the statistical analysis indicated that both mechanical and physical properties were significantly influenced by the investigated variables.

**Table A1:** ANOVAs of the variable parameters on the physical properties of plywood panels.

Variables	Parameter	SS	df	MS	F	Sig.
A	Thickness	0.516	3	0.172	43.273	0.000
	Density	5333.171	3	1777.724	3.371	0.021
	TS (2 h)	301.708	3	100.569	110.812	0.000
	TS (24 h)	596.543	3	198.848	90.455	0.000
	WA (2 h)	3138.794	3	1046.265	66.364	0.000
	WA (24 h)	5973.605	3	1991.202	112.867	0.000
W	Thickness	1.757	2	0.878	220.842	0.000
	Density	1,382,999.814	2	691,499.907	1311.200	0.000
	TS (2 h)	111.794	2	55.897	61.590	0.000
	TS (24 h)	124.645	2	62.323	28.350	0.000
	WA (2 h)	1518.766	2	759.383	48.167	0.000
	WA (24 h)	2798.687	2	1399.344	79.319	0.000
A × W	Thickness	0.599	6	0.100	25.117	0.000
	Density	21,883.164	6	3647.194	6.916	0.000
	TS (2 h)	69.138	6	11.523	12.697	0.000
	TS (24 h)	196.978	5	39.396	17.921	0.000
	WA (2 h)	876.147	6	146.025	9.262	0.000
	WA (24 h)	1162.316	6	193.719	10.981	0.000

Note: A = type of adhesive; W = wood species; TS (2 h) = thickness swelling in 2 h; TS (24 h) = thickness swelling in 24 h; WA (2 h) = water absorption in 2 h; WA (24 h) = water absorption in 24 h; SS = sum of squares; df = degrees of freedom; MS = mean square; F = *F* value; Sig. = significance probability (*p*-value). Significant difference at the 5% level (*p* ≤ 0.05).

**Table A2:** ANOVAs of the variable parameters on the mechanical properties of plywood panels.

Variables	Parameter	SS	df	MS	F	Sig.
A	MOR	25,068.998	3	8356.333	137.170	0.000
	MOE	9,070,565.122	3	3,023,521.707	3.124	0.032
	TSS	602.642	3	200.881	553.556	0.000
	ShS	26.667	3	8.889	346.858	0.000
W	MOR	15,813.783	2	7906.891	129.792	0.000
	MOE	2.173 × 10^8^	2	1.087 × 10^8^	112.275	0.000
	TSS	111.563	2	55.781	153.714	0.000
	ShS	10.978	2	5.489	214.178	0.000
A × W	MOR	15,236.552	6	2539.425	41.685	0.000
	MOE	54,482,070.823	6	9,080,345.137	9.382	0.000
	TSS	100.071	6	16.678	45.960	0.000
	ShS	10.293	6	1.716	66.940	0.000

Note: A = type of adhesive; W = wood species; MOR = bending strength; MOE = modulus of elasticity in bending; TSS = tensile shear strength; ShS = shear strength; SS = sum of squares; df = degrees of freedom; MS = mean square; F = *F* value; Sig. = significance probability (*p*-value). Significant difference at the 5% level (*p* ≤ 0.05).

**Table A3:** Duncan’s test results for selected physical and mechanical properties of plywood panels. *

Variable	Thickness (mm)	Density (kg/m^3^)	MOR (MPa)	MOE (MPa)	ShS (MPa)	TSS (MPa)	TS (24 h) (%)	WA (24 h) (%)
Adhesive:								
MUF	9.06 ± 0.04 c	627.49 ± 124.89 c	99.81 ± 20.00 b	13,014.88 ± 1767.26 a	2.02 ± 0.36 c	10.17 ± 1.97 d	7.25 ± 1.70 a	32.80 ± 5.36 a
LDPE	8.97 ± 0.18 b	612.26 ± 130.82 ab	72.28 ± 12.74 a	12,959.76 ± 1436.76 a	1.18 ± 0.14 a	4.52 ± 0.59 a	12.88 ± 1.92 c	52.90 ± 7.58 c
CoPA	8.91 ± 0.15 a	622.11 ± 115.85 bc	112.18 ± 27.20 c	13,248.43 ± 3113.24 ab	2.51 ± 0.83 d	7.70 ± 0.70 b	11.30 ± 2.94 b	42.49 ± 7.56 b
CoPE	8.89 ± 0.21 a	609.01 ± 112.98 a	116.10 ± 23.41 c	13,819.41 ± 1950.06 b	1.78 ± 0.21 b	9.29 ± 1.87 c	12.35 ± 2.00 c	43.48 ± 9.71 b
Wood species:								
Beech	9.08 ± 0.02 c	715.79 ± 8.76 c	109.91 ± 33.61 b	14,167.62 ± 947.03 b	2.16 ± 0.72 b	8.40 ± 2.54 b	11.20 ± 2.85 b	48.65 ± 7.80 c
Birch	8.99 ± 0.09 b	665.05 ± 22.69 b	109.37 ± 23.34 b	14,792.66 ± 1236.69 c	2.10 ± 0.69 b	8.58 ± 2.94 b	11.46 ± 2.46 b	35.89 ± 10.08 a
Spruce	8.81 ± 0.16 a	483.22 ± 11.20 a	79.51 ± 7.26 a	11,028.88 ± 1627.14 a	1.40 ± 0.26 a	6.62 ± 1.57 a	9.63 ± 3.58 a	41.75 ± 8.56 b

* Different letters a–d denotes a significant difference. The means followed by the same letter do not statistically differ from each other (*p* ≤ 0.05).
